# Methyl­naltrexone bromide methanol monosolvate

**DOI:** 10.1107/S1600536812005545

**Published:** 2012-02-24

**Authors:** Guoqing Li, Xu Cai, Zhibing Zheng, Xinbo Zhou, Song Li

**Affiliations:** a Shenyang Pharmaceutical University, Liaoning 110016, People’s Republic of China; b Beijing Institute of Pharmacology and Toxicology, Beijing 100850, People’s Republic of China

## Abstract

In the title compound [systematic name: (4*R*,4a*S*,7a*R*,12b*S*)-3-cyclo­propyl­meth­yl-4a,9-hy­droxy-7-oxo-2,3,4,4a,5,6,7,7a-octa­hydro-1*H*-4,12-methano­benzofuro[3,2-*e*]isoquinolin-3-ium bromide methanol monosolvate], C_21_H_26_NO_4_^+^·Br^−^·CH_3_OH, two of the three six-membered rings adopt chair conformations while the third, which contains a C=C double bond, adopts an approximate half-boat conformation. The 2,3-dihydro­furan ring adopts an envelope conformation. In the crystal, the components are linked by O—H⋯O and O—H⋯Br hydrogen bonds. The absolute stereochemistry was inferred from one of the starting materials.

## Related literature

For general background to methyl­naltrexone (MNTX) bromide, see: Crabtree (1984 ▶). For the bioactivity and synthesis of *R*-MNTX bromide, see: Baker (2009 ▶); Doshan & Perez (2006 ▶).
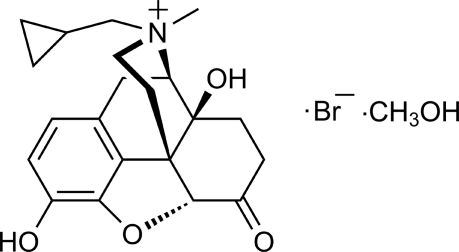


## Experimental

### 

#### Crystal data

C_21_H_26_NO_4_^+^·Br^−^·CH_4_O
*M* *_r_* = 468.38
Orthorhombic, 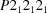

*a* = 7.3335 (11) Å
*b* = 12.956 (2) Å
*c* = 21.506 (3) Å
*V* = 2043.4 (5) Å^3^
*Z* = 4
Mo *K*α radiation
μ = 2.05 mm^−1^
*T* = 113 K
0.20 × 0.18 × 0.12 mm

#### Data collection

Rigaku Saturn CCD area-detector diffractometer
Absorption correction: multi-scan (*CrystalClear*; Rigaku/MSC, 2005 ▶) *T*_min_ = 0.685, *T*_max_ = 0.791
15852 measured reflections
4179 independent reflections
3287 reflections with *I* > 2σ(*I*)
*R*_int_ = 0.042

#### Refinement

*R*[*F* ^2^ > 2σ(*F* ^2^)] = 0.024
*wR*(*F* ^2^) = 0.047
*S* = 0.90
4179 reflections
267 parameters
H-atom parameters constrained
Δρ_max_ = 0.41 e Å^−3^
Δρ_min_ = −0.23 e Å^−3^
Absolute structure: Flack (1983 ▶), 1768 Friedel pairs
Flack parameter: 0.010 (6)

### 

Data collection: *CrystalClear* (Rigaku/MSC, 2005 ▶); cell refinement: *CrystalClear*; data reduction: *CrystalClear*; program(s) used to solve structure: *SHELXS97* (Sheldrick, 2008 ▶); program(s) used to refine structure: *SHELXL97* (Sheldrick, 2008 ▶); molecular graphics: *SHELXTL* (Sheldrick, 2008 ▶); software used to prepare material for publication: *SHELXTL*.

## Supplementary Material

Crystal structure: contains datablock(s) I, global. DOI: 10.1107/S1600536812005545/mw2034sup1.cif

Structure factors: contains datablock(s) I. DOI: 10.1107/S1600536812005545/mw2034Isup2.hkl

Additional supplementary materials:  crystallographic information; 3D view; checkCIF report

## Figures and Tables

**Table 1:** Hydrogen-bond geometry (Å, °)

*D*—H⋯*A*	*D*—H	H⋯*A*	*D*⋯*A*	*D*—H⋯*A*
O1—H1⋯O5^i^	0.84	1.78	2.613 (2)	170
O4—H4⋯Br1	0.84	2.39	3.2272 (16)	175
O5—H5⋯Br1^ii^	0.84	2.42	3.2376 (16)	165

Symmetry codes: (i) 
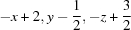
; (ii) 
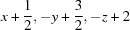
.

**Fig. 1.**
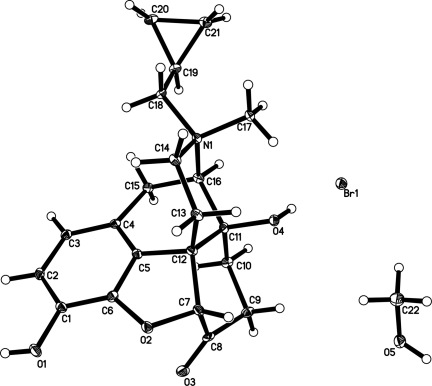
The molecular structure of the title compound, with 50% displacement ellipsoids.

**Fig. 2.**
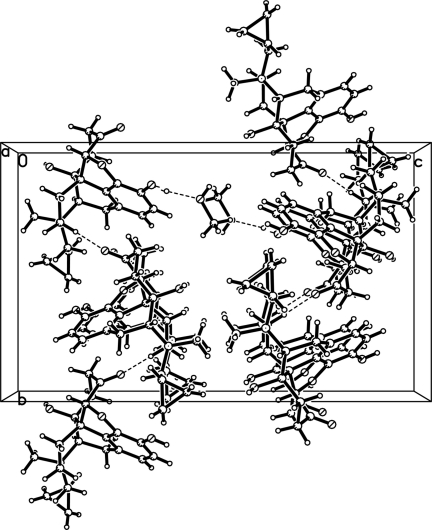
Packing of the title compound viewed down the a axis showing the C—H···O interactions.

## supplementary crystallographic information

### Comment

Methylnaltrexone (MNTX) is a quaternary derivative of the pure opioid antagonist naltrexone, which has greater polarity and lower lipid solubility and does not cross the blood-brain barrier in humans thus enabling reversal of opioid-induced peripheral effects such as constipation without affecting the central effects such as pain relief (Baker, 2009). *R*-MNTX bromide has been used in clinical applications to counteract addiction caused by meconium drugs. This paper reports the synthesis and crystal structure of the title compound *R*-MNTX bromide methanol solvate.

In the title compound, C_21_H_26_BrNO_4_.CH_3_OH, (Fig.1), two of the three six-membered rings adopt chair conformations while the third, which contains a C=C double bond, adopts an approximate half-boat conformation. The 2,3-dihydrofuran ring adopts an envelope conformation. The structure displays *O*—*H*···O hydrogen bonding (Table 1, Fig. 2).

### Experimental

A solution of (4*R*,4a*S*,7aR*R*,12b*S*)-3-(cyclopropylmethyl)-4a,9-dihydroxy-2,3,4,4a,5,6-hexahydro-1*H*-4,12-methanobenzofuro[3,2-*e*]isoquinolin-7(7a*H*)-one (1 g, 2.94 mmol) and MeI (3.4 g) in 1-methyl-2-pyrrolidinone (12 ml) was stirred at 140 K for 10 h and the solvent was removed under reduced pressure. The residue was dissolved in water and the pH was adjusted to about 9 with 10% Na_2_CO_3_ solution and after filtration the crude solid was obtained. Colorless single crystals of the title compound were obtained by recrystallization from a methanol–water solution acidified with HBr_aq_. Anal. Calc. (%) for C_21_H_26_BrNO_4_: C, 57.81; H, 6.01; Br, 18.31; N, 3.21. Found: C, 58.00; H, 6.11; Br, 18.30; N, 3.25.

### Refinement

All H atoms were placed in calculated positions with C—H distances ranging from 0.95 to 1.00 Å and included in the refinement in the riding-model approximation with *U*_iso_ = 1.2Ueq (1.5Ueq for methyl) of the attached atom. The absolute configuration was inferred from that of the starting material.

### Crystal data

**Table:**

C_21_H_26_NO_4_^+^·Br^−^·CH_4_O	*F*(000) = 976
*M**_r_* = 468.38	*D*_x_ = 1.523 Mg m^−^^3^
Orthorhombic, *P*2_1_2_1_2_1_	Mo *K*α radiation, λ = 0.71073 Å
Hall symbol: P 2ac 2ab	Cell parameters from 7107 reflections
*a* = 7.3335 (11) Å	θ = 1.6–27.9°
*b* = 12.956 (2) Å	µ = 2.05 mm^−^^1^
*c* = 21.506 (3) Å	*T* = 113 K
*V* = 2043.4 (5) Å^3^	Prism, colorless
*Z* = 4	0.20 × 0.18 × 0.12 mm

### Data collection

**Table:**

Rigaku Saturn CCD area-detector diffractometer	4179 independent reflections
Radiation source: fine-focus sealed tube	3287 reflections with *I* > 2σ(*I*)
Graphite monochromator	*R*_int_ = 0.042
Detector resolution: 14.63 pixels mm^-1^	θ_max_ = 26.4°, θ_min_ = 1.8°
ω and φ scans	*h* = −9→8
Absorption correction: multi-scan (*CrystalClear*; Rigaku/MSC, 2005)	*k* = −16→15
*T*_min_ = 0.685, *T*_max_ = 0.791	*l* = −23→26
15852 measured reflections	

### Refinement

**Table:**

Refinement on *F*^2^	Secondary atom site location: difference Fourier map
Least-squares matrix: full	Hydrogen site location: inferred from neighbouring sites
*R*[*F*^2^ > 2σ(*F*^2^)] = 0.024	H-atom parameters constrained
*wR*(*F*^2^) = 0.047	*w* = 1/[σ^2^(*F*_o_^2^) + (0.0141*P*)^2^] where *P* = (*F*_o_^2^ + 2*F*_c_^2^)/3
*S* = 0.90	(Δ/σ)_max_ = 0.001
4179 reflections	Δρ_max_ = 0.41 e Å^−^^3^
267 parameters	Δρ_min_ = −0.23 e Å^−^^3^
0 restraints	Absolute structure: Flack (1983), 1768 Friedel pairs
Primary atom site location: structure-invariant direct methods	Flack parameter: 0.010 (6)

### Special details

**Table:**

Geometry. All esds (except the esd in the dihedral angle between two l.s. planes) are estimated using the full covariance matrix. The cell esds are taken into account individually in the estimation of esds in distances, angles and torsion angles; correlations between esds in cell parameters are only used when they are defined by crystal symmetry. An approximate (isotropic) treatment of cell esds is used for estimating esds involving l.s. planes.
Refinement. Refinement of F^2^ against ALL reflections. The weighted R-factor wR and goodness of fit S are based on F^2^, conventional R-factors R are based on F, with F set to zero for negative F^2^. The threshold expression of F^2^ > 2sigma(F^2^) is used only for calculating R-factors(gt) etc. and is not relevant to the choice of reflections for refinement. R-factors based on F^2^ are statistically about twice as large as those based on F, and R- factors based on ALL data will be even larger.

### Fractional atomic coordinates and isotropic or equivalent isotropic displacement parameters (Å^2^)

**Table:**

	*x*	*y*	*z*	*U*_iso_*/*U*_eq_	
Br1	0.29599 (3)	0.501662 (19)	0.961583 (10)	0.02197 (6)	
O1	1.2501 (2)	0.34387 (12)	0.65333 (8)	0.0234 (4)	
H1	1.2547	0.3189	0.6173	0.035*	
O2	1.1484 (2)	0.42585 (12)	0.77342 (7)	0.0182 (4)	
O3	0.9456 (2)	0.58534 (12)	0.73226 (8)	0.0238 (4)	
O4	0.6957 (2)	0.43437 (11)	0.91369 (7)	0.0165 (4)	
H4	0.5895	0.4480	0.9258	0.025*	
N1	0.6665 (2)	0.21015 (13)	0.88781 (9)	0.0144 (5)	
C1	1.0882 (3)	0.31810 (16)	0.68032 (11)	0.0164 (6)	
C2	0.9548 (3)	0.25521 (16)	0.65245 (11)	0.0174 (6)	
H2	0.9779	0.2273	0.6124	0.021*	
C3	0.7903 (3)	0.23257 (15)	0.68151 (10)	0.0164 (5)	
H3	0.7021	0.1918	0.6605	0.020*	
C4	0.7533 (3)	0.26886 (15)	0.74100 (11)	0.0142 (5)	
C5	0.8893 (3)	0.32771 (16)	0.76833 (11)	0.0135 (5)	
C6	1.0473 (3)	0.35531 (17)	0.73832 (11)	0.0158 (5)	
C7	1.0143 (3)	0.46915 (16)	0.81590 (11)	0.0170 (6)	
H7	1.0740	0.4969	0.8542	0.020*	
C8	0.9052 (3)	0.55228 (17)	0.78292 (11)	0.0180 (6)	
C9	0.7271 (3)	0.58009 (16)	0.81494 (11)	0.0201 (6)	
H9A	0.6685	0.6394	0.7938	0.024*	
H9B	0.7494	0.5988	0.8589	0.024*	
C10	0.6045 (3)	0.48386 (16)	0.81087 (11)	0.0171 (6)	
H10A	0.4805	0.5003	0.8261	0.021*	
H10B	0.5951	0.4604	0.7672	0.021*	
C11	0.6899 (3)	0.39893 (15)	0.85102 (11)	0.0144 (5)	
C12	0.8869 (3)	0.37669 (16)	0.83171 (11)	0.0143 (5)	
C13	0.9708 (3)	0.30462 (16)	0.88064 (11)	0.0164 (5)	
H13A	1.1013	0.2933	0.8710	0.020*	
H13B	0.9629	0.3374	0.9221	0.020*	
C14	0.8727 (3)	0.20165 (16)	0.88195 (11)	0.0163 (5)	
H14A	0.9196	0.1607	0.9174	0.020*	
H14B	0.9019	0.1635	0.8433	0.020*	
C15	0.5739 (3)	0.25964 (17)	0.77574 (10)	0.0153 (6)	
H15A	0.5331	0.1868	0.7743	0.018*	
H15B	0.4810	0.3018	0.7541	0.018*	
C16	0.5843 (3)	0.29442 (16)	0.84432 (11)	0.0144 (5)	
H16	0.4564	0.3071	0.8585	0.017*	
C17	0.6162 (3)	0.23066 (16)	0.95469 (11)	0.0188 (6)	
H17A	0.6519	0.1715	0.9803	0.028*	
H17B	0.4842	0.2412	0.9579	0.028*	
H17C	0.6798	0.2926	0.9693	0.028*	
C18	0.5965 (3)	0.10122 (15)	0.87173 (11)	0.0172 (6)	
H18A	0.6365	0.0839	0.8290	0.021*	
H18B	0.6545	0.0512	0.9003	0.021*	
C19	0.3938 (3)	0.08794 (16)	0.87549 (11)	0.0181 (6)	
H19	0.3178	0.1369	0.8506	0.022*	
C20	0.3307 (3)	−0.02288 (15)	0.87791 (11)	0.0210 (6)	
H20A	0.2193	−0.0414	0.8544	0.025*	
H20B	0.4252	−0.0774	0.8794	0.025*	
C21	0.3093 (4)	0.04501 (16)	0.93415 (11)	0.0220 (6)	
H21A	0.3906	0.0321	0.9701	0.026*	
H21B	0.1847	0.0682	0.9451	0.026*	
O5	0.7189 (2)	0.78677 (11)	0.96273 (8)	0.0307 (4)	
H5	0.7586	0.8364	0.9839	0.046*	
C22	0.7505 (3)	0.69215 (17)	0.99537 (12)	0.0289 (7)	
H22A	0.8228	0.6456	0.9693	0.043*	
H22B	0.6334	0.6596	1.0052	0.043*	
H22C	0.8168	0.7065	1.0340	0.043*	

### Atomic displacement parameters (Å^2^)

**Table:**

	*U*^11^	*U*^22^	*U*^33^	*U*^12^	*U*^13^	*U*^23^
Br1	0.02033 (12)	0.02373 (11)	0.02185 (12)	−0.00216 (15)	0.00200 (12)	−0.00233 (15)
O1	0.0236 (12)	0.0294 (9)	0.0171 (10)	−0.0052 (8)	0.0075 (8)	−0.0035 (8)
O2	0.0148 (9)	0.0231 (9)	0.0169 (10)	−0.0047 (7)	0.0025 (8)	−0.0034 (7)
O3	0.0346 (11)	0.0213 (9)	0.0155 (11)	−0.0085 (8)	−0.0031 (9)	0.0025 (8)
O4	0.0165 (9)	0.0177 (8)	0.0153 (9)	−0.0016 (8)	0.0013 (8)	−0.0026 (7)
N1	0.0169 (12)	0.0148 (10)	0.0114 (11)	−0.0002 (8)	0.0007 (9)	0.0000 (8)
C1	0.0150 (14)	0.0170 (13)	0.0172 (15)	0.0022 (10)	0.0027 (11)	0.0026 (11)
C2	0.0275 (15)	0.0153 (12)	0.0093 (14)	0.0040 (11)	0.0002 (12)	0.0001 (10)
C3	0.0191 (13)	0.0142 (11)	0.0158 (14)	−0.0027 (11)	−0.0030 (13)	−0.0005 (9)
C4	0.0161 (15)	0.0121 (11)	0.0144 (13)	0.0006 (9)	−0.0013 (10)	0.0029 (9)
C5	0.0157 (13)	0.0138 (12)	0.0111 (14)	0.0020 (10)	−0.0003 (11)	0.0027 (10)
C6	0.0154 (14)	0.0162 (12)	0.0157 (14)	−0.0021 (10)	−0.0036 (12)	−0.0016 (10)
C7	0.0162 (13)	0.0227 (15)	0.0121 (14)	−0.0058 (10)	0.0005 (11)	−0.0005 (10)
C8	0.0202 (15)	0.0163 (13)	0.0176 (16)	−0.0120 (11)	−0.0052 (12)	−0.0024 (11)
C9	0.0256 (15)	0.0139 (12)	0.0208 (14)	−0.0021 (11)	−0.0072 (13)	0.0042 (10)
C10	0.0141 (12)	0.0189 (15)	0.0183 (13)	−0.0018 (11)	−0.0052 (11)	0.0007 (11)
C11	0.0177 (13)	0.0163 (12)	0.0092 (13)	0.0001 (11)	−0.0029 (12)	0.0000 (9)
C12	0.0132 (13)	0.0175 (13)	0.0121 (14)	−0.0032 (11)	−0.0008 (11)	0.0013 (10)
C13	0.0136 (13)	0.0215 (13)	0.0142 (14)	0.0001 (10)	−0.0005 (11)	0.0031 (11)
C14	0.0154 (13)	0.0201 (13)	0.0133 (14)	0.0018 (11)	−0.0021 (11)	0.0024 (11)
C15	0.0164 (14)	0.0168 (13)	0.0128 (14)	−0.0034 (10)	−0.0038 (12)	0.0008 (10)
C16	0.0099 (13)	0.0170 (12)	0.0162 (14)	0.0023 (10)	−0.0004 (11)	0.0027 (10)
C17	0.0260 (14)	0.0182 (12)	0.0122 (14)	0.0003 (11)	0.0047 (13)	−0.0005 (11)
C18	0.0239 (15)	0.0102 (12)	0.0174 (15)	−0.0020 (10)	0.0025 (12)	−0.0007 (10)
C19	0.0184 (14)	0.0143 (12)	0.0217 (16)	−0.0016 (11)	−0.0004 (12)	0.0023 (11)
C20	0.0199 (14)	0.0162 (15)	0.0269 (15)	−0.0058 (10)	−0.0042 (11)	0.0027 (10)
C21	0.0221 (14)	0.0217 (12)	0.0222 (15)	−0.0019 (11)	0.0024 (13)	0.0037 (10)
O5	0.0504 (12)	0.0223 (9)	0.0193 (10)	−0.0031 (9)	0.0040 (11)	−0.0007 (8)
C22	0.0353 (18)	0.0246 (13)	0.0267 (16)	−0.0003 (11)	0.0002 (13)	0.0039 (11)

### Geometric parameters (Å, º)

**Table:**

O1—C1	1.363 (3)	C11—C12	1.531 (3)
O1—H1	0.8400	C11—C16	1.567 (3)
O2—C6	1.398 (3)	C12—C13	1.536 (3)
O2—C7	1.455 (3)	C13—C14	1.516 (3)
O3—C8	1.208 (3)	C13—H13A	0.9900
O4—C11	1.424 (2)	C13—H13B	0.9900
O4—H4	0.8400	C14—H14A	0.9900
N1—C17	1.508 (3)	C14—H14B	0.9900
N1—C14	1.521 (3)	C15—C16	1.544 (3)
N1—C18	1.541 (3)	C15—H15A	0.9900
N1—C16	1.559 (3)	C15—H15B	0.9900
C1—C6	1.370 (3)	C16—H16	1.0000
C1—C2	1.407 (3)	C17—H17A	0.9800
C2—C3	1.390 (3)	C17—H17B	0.9800
C2—H2	0.9500	C17—H17C	0.9800
C3—C4	1.390 (3)	C18—C19	1.499 (3)
C3—H3	0.9500	C18—H18A	0.9900
C4—C5	1.386 (3)	C18—H18B	0.9900
C4—C15	1.517 (3)	C19—C20	1.509 (3)
C5—C6	1.374 (3)	C19—C21	1.512 (3)
C5—C12	1.503 (3)	C19—H19	1.0000
C7—C8	1.518 (3)	C20—C21	1.504 (3)
C7—C12	1.557 (3)	C20—H20A	0.9900
C7—H7	1.0000	C20—H20B	0.9900
C8—C9	1.520 (3)	C21—H21A	0.9900
C9—C10	1.539 (3)	C21—H21B	0.9900
C9—H9A	0.9900	O5—C22	1.432 (2)
C9—H9B	0.9900	O5—H5	0.8400
C10—C11	1.533 (3)	C22—H22A	0.9800
C10—H10A	0.9900	C22—H22B	0.9800
C10—H10B	0.9900	C22—H22C	0.9800
			
C1—O1—H1	109.5	C14—C13—H13A	109.4
C6—O2—C7	103.46 (16)	C12—C13—H13A	109.4
C11—O4—H4	109.5	C14—C13—H13B	109.4
C17—N1—C14	109.55 (18)	C12—C13—H13B	109.4
C17—N1—C18	107.10 (17)	H13A—C13—H13B	108.0
C14—N1—C18	104.24 (17)	C13—C14—N1	114.19 (19)
C17—N1—C16	110.75 (16)	C13—C14—H14A	108.7
C14—N1—C16	112.65 (17)	N1—C14—H14A	108.7
C18—N1—C16	112.23 (17)	C13—C14—H14B	108.7
O1—C1—C6	119.5 (2)	N1—C14—H14B	108.7
O1—C1—C2	124.5 (2)	H14A—C14—H14B	107.6
C6—C1—C2	116.0 (2)	C4—C15—C16	113.86 (18)
C3—C2—C1	122.3 (2)	C4—C15—H15A	108.8
C3—C2—H2	118.9	C16—C15—H15A	108.8
C1—C2—H2	118.9	C4—C15—H15B	108.8
C2—C3—C4	120.8 (2)	C16—C15—H15B	108.8
C2—C3—H3	119.6	H15A—C15—H15B	107.7
C4—C3—H3	119.6	C15—C16—N1	112.82 (17)
C5—C4—C3	115.9 (2)	C15—C16—C11	111.37 (18)
C5—C4—C15	117.3 (2)	N1—C16—C11	111.05 (17)
C3—C4—C15	126.6 (2)	C15—C16—H16	107.1
C6—C5—C4	123.4 (2)	N1—C16—H16	107.1
C6—C5—C12	109.0 (2)	C11—C16—H16	107.1
C4—C5—C12	127.5 (2)	N1—C17—H17A	109.5
C1—C6—C5	121.4 (2)	N1—C17—H17B	109.5
C1—C6—O2	127.3 (2)	H17A—C17—H17B	109.5
C5—C6—O2	111.3 (2)	N1—C17—H17C	109.5
O2—C7—C8	109.7 (2)	H17A—C17—H17C	109.5
O2—C7—C12	104.24 (17)	H17B—C17—H17C	109.5
C8—C7—C12	109.39 (19)	C19—C18—N1	115.04 (18)
O2—C7—H7	111.1	C19—C18—H18A	108.5
C8—C7—H7	111.1	N1—C18—H18A	108.5
C12—C7—H7	111.1	C19—C18—H18B	108.5
O3—C8—C7	123.0 (2)	N1—C18—H18B	108.5
O3—C8—C9	122.4 (2)	H18A—C18—H18B	107.5
C7—C8—C9	114.2 (2)	C18—C19—C20	114.5 (2)
C8—C9—C10	106.51 (18)	C18—C19—C21	119.6 (2)
C8—C9—H9A	110.4	C20—C19—C21	59.71 (14)
C10—C9—H9A	110.4	C18—C19—H19	116.8
C8—C9—H9B	110.4	C20—C19—H19	116.8
C10—C9—H9B	110.4	C21—C19—H19	116.8
H9A—C9—H9B	108.6	C21—C20—C19	60.22 (14)
C11—C10—C9	108.12 (18)	C21—C20—H20A	117.7
C11—C10—H10A	110.1	C19—C20—H20A	117.7
C9—C10—H10A	110.1	C21—C20—H20B	117.7
C11—C10—H10B	110.1	C19—C20—H20B	117.7
C9—C10—H10B	110.1	H20A—C20—H20B	114.9
H10A—C10—H10B	108.4	C20—C21—C19	60.07 (14)
O4—C11—C12	106.82 (18)	C20—C21—H21A	117.8
O4—C11—C10	108.29 (17)	C19—C21—H21A	117.8
C12—C11—C10	111.59 (19)	C20—C21—H21B	117.8
O4—C11—C16	112.36 (19)	C19—C21—H21B	117.8
C12—C11—C16	106.19 (17)	H21A—C21—H21B	114.9
C10—C11—C16	111.52 (18)	C22—O5—H5	109.5
C5—C12—C11	109.64 (19)	O5—C22—H22A	109.5
C5—C12—C13	111.10 (18)	O5—C22—H22B	109.5
C11—C12—C13	107.86 (19)	H22A—C22—H22B	109.5
C5—C12—C7	96.89 (19)	O5—C22—H22C	109.5
C11—C12—C7	118.74 (19)	H22A—C22—H22C	109.5
C13—C12—C7	112.16 (19)	H22B—C22—H22C	109.5
C14—C13—C12	110.95 (19)		
			
O1—C1—C2—C3	−178.9 (2)	C10—C11—C12—C13	170.80 (17)
C6—C1—C2—C3	0.5 (3)	C16—C11—C12—C13	−67.5 (2)
C1—C2—C3—C4	−2.2 (3)	O4—C11—C12—C7	−76.3 (3)
C2—C3—C4—C5	0.0 (3)	C10—C11—C12—C7	41.8 (3)
C2—C3—C4—C15	173.4 (2)	C16—C11—C12—C7	163.6 (2)
C3—C4—C5—C6	4.0 (3)	O2—C7—C12—C5	−36.9 (2)
C15—C4—C5—C6	−170.0 (2)	C8—C7—C12—C5	80.3 (2)
C3—C4—C5—C12	180.0 (2)	O2—C7—C12—C11	−153.83 (19)
C15—C4—C5—C12	5.9 (3)	C8—C7—C12—C11	−36.6 (3)
O1—C1—C6—C5	−177.2 (2)	O2—C7—C12—C13	79.2 (2)
C2—C1—C6—C5	3.4 (3)	C8—C7—C12—C13	−163.55 (19)
O1—C1—C6—O2	5.4 (4)	C5—C12—C13—C14	−56.7 (3)
C2—C1—C6—O2	−174.0 (2)	C11—C12—C13—C14	63.5 (2)
C4—C5—C6—C1	−5.9 (4)	C7—C12—C13—C14	−163.96 (19)
C12—C5—C6—C1	177.5 (2)	C12—C13—C14—N1	−51.3 (3)
C4—C5—C6—O2	171.89 (19)	C17—N1—C14—C13	−79.4 (2)
C12—C5—C6—O2	−4.7 (3)	C18—N1—C14—C13	166.29 (19)
C7—O2—C6—C1	157.3 (2)	C16—N1—C14—C13	44.4 (3)
C7—O2—C6—C5	−20.3 (2)	C5—C4—C15—C16	−14.7 (3)
C6—O2—C7—C8	−80.8 (2)	C3—C4—C15—C16	172.0 (2)
C6—O2—C7—C12	36.2 (2)	C4—C15—C16—N1	−80.4 (2)
O2—C7—C8—O3	−9.7 (3)	C4—C15—C16—C11	45.3 (3)
C12—C7—C8—O3	−123.5 (2)	C17—N1—C16—C15	−160.55 (18)
O2—C7—C8—C9	162.42 (17)	C14—N1—C16—C15	76.4 (2)
C12—C7—C8—C9	48.7 (3)	C18—N1—C16—C15	−40.9 (2)
O3—C8—C9—C10	106.3 (2)	C17—N1—C16—C11	73.6 (2)
C7—C8—C9—C10	−65.9 (2)	C14—N1—C16—C11	−49.5 (2)
C8—C9—C10—C11	67.1 (2)	C18—N1—C16—C11	−166.77 (18)
C9—C10—C11—O4	61.6 (2)	O4—C11—C16—C15	177.95 (18)
C9—C10—C11—C12	−55.7 (2)	C12—C11—C16—C15	−65.6 (2)
C9—C10—C11—C16	−174.32 (19)	C10—C11—C16—C15	56.2 (2)
C6—C5—C12—C11	149.25 (19)	O4—C11—C16—N1	−55.4 (2)
C4—C5—C12—C11	−27.2 (3)	C12—C11—C16—N1	61.0 (2)
C6—C5—C12—C13	−91.6 (2)	C10—C11—C16—N1	−177.20 (18)
C4—C5—C12—C13	92.0 (3)	C17—N1—C18—C19	62.6 (2)
C6—C5—C12—C7	25.4 (2)	C14—N1—C18—C19	178.7 (2)
C4—C5—C12—C7	−151.1 (2)	C16—N1—C18—C19	−59.1 (3)
O4—C11—C12—C5	173.74 (16)	N1—C18—C19—C20	−163.38 (19)
C10—C11—C12—C5	−68.1 (2)	N1—C18—C19—C21	−95.6 (2)
C16—C11—C12—C5	53.6 (2)	C18—C19—C20—C21	111.3 (2)
O4—C11—C12—C13	52.6 (2)	C18—C19—C21—C20	−102.8 (2)

### Hydrogen-bond geometry (Å, º)

**Table:**

*D*—H···*A*	*D*—H	H···*A*	*D*···*A*	*D*—H···*A*
O1—H1···O5^i^	0.84	1.78	2.613 (2)	170
O4—H4···Br1	0.84	2.39	3.2272 (16)	175
O5—H5···Br1^ii^	0.84	2.42	3.2376 (16)	165

Symmetry codes: (i) −*x*+2, *y*−1/2, −*z*+3/2; (ii) *x*+1/2, −*y*+3/2, −*z*+2.
